# Security Analysis and Improvement of an Image Encryption Cryptosystem Based on Bit Plane Extraction and Multi Chaos

**DOI:** 10.3390/e23050505

**Published:** 2021-04-22

**Authors:** Shuqin Zhu, Congxu Zhu

**Affiliations:** 1 School of Computer Science, Liaocheng University, Liaocheng 252059, China; shuqinzhu2008@163.com; 2 School of Computer Science and Engineering, Central South University, Changsha 410083, China

**Keywords:** information security, image encryption, security analysis, chosen-plaintext attack

## Abstract

This paper analyzes the security of image encryption systems based on bit plane extraction and multi chaos. It includes a bit-level permutation for high, 4-bit planes and bit-wise XOR diffusion, and finds that the key streams in the permutation and diffusion phases are independent of the plaintext image. Therefore, the equivalent diffusion key and the equivalent permutation key can be recovered by the chosen-plaintext attack method, in which only two special plaintext images and their corresponding cipher images are used. The effectiveness and feasibility of the proposed attack algorithm is verified by a MATLAB 2015b simulation. In the experiment, all the key streams in the original algorithm are cracked through two special plaintext images and their corresponding ciphertext images. In addition, an improved algorithm is proposed. In the improved algorithm, the generation of a random sequence is related to ciphertext, which makes the encryption algorithm have the encryption effect of a “one time pad”. The encryption effect of the improved algorithm is better than that of the original encryption algorithm in the aspects of information entropy, ciphertext correlation analysis and ciphertext sensitivity analysis.

## 1. Introduction

With the rapid development of computer and internet technology, all kinds of multimedia data including digital images are transmitted through a network and stored on a disk, which greatly facilitates people’s work and life. Image information can easily be illegally copied, tampered with, spread, and used for other malicious damage in the process of image transmission. Therefore, it is necessary to adopt reliable image encryption technology to ensure the safe transmission and storage of digital images. As described in [1], the main techniques used in image encryption algorithms include chaotic mapping, DNA computing, neural networks, compressed sensing, cellular automata, wavelet transformation, and so on. However, chaos has become an ideal tool for designing secure and efficient encryption schemes due to the sensitivity, ergodicity and randomness of chaotic systems under the initial conditions and the system parameters, which coincides with the two basic principles of cryptography: diffusion and confusion. In 1998, Friedrich [2] proposed an alternative diffusion encryption architecture, which was later developed into a classical scrambling diffusion encryption architecture [3,4,5]. Based on this structure, scholars have proposed many image encryption algorithms [6,7,8,9,10,11,12,13,14]. Chai et al. [6] designed a color image encryption algorithm based on a four-wing hyperchaotic system and DNA coding. The generation of random sequences and DNA coding sequences used in the algorithm is related to plaintext. Wang et al. [7] proposed a new image encryption algorithm in which the cipher pixel value depends on two random, nonadjacent pixels and a chaos interference value. A new chaos-based image encryption algorithm was designed by Li et al. [8], which adopts the orbit perturbation and the dynamic state variable selection mechanisms. Zhu et al. [9] constructed a five-dimensional, discrete, hyper-chaotic map by combining the logistic map and the 3D discrete Lorenz map, and designed a block-based image encryption scheme related to a plain image based on this chaotic system. A chaotic image encryption, using the Hopfield model and Hindmarsh–Rose neurons implemented on FPGA, was presented in [15], which was focused on finding suitable coefficient values of neurons to generate robust random binary sequences that can be used in image encryption. In [16], a new algorithm to improve the randomness of five chaotic maps that were implemented on a PIC micro-controller was proposed. The improved chaotic maps were tested to encrypt digital images in a wireless communication scheme, particularly on a machine to machine (M2M) link, via ZigBee channels.

The operation of the above algorithms was based on the pixel level. At the same time, the chaotic image encryption algorithm based on the bit-level technique has also attracted the attention of researchers due to its reliability and effectiveness [17,18,19]. Wang et al. [17] proposed a hyperchaos-based image encryption algorithm based on bit-level permutation and DNA encoding. In [18], a symmetric color image encryption algorithm adopting bit-permutation was presented, in which the key streams are closely related to the plain image. In 2018, an image encryption algorithm with an avalanche effect based on bit-level substitution was proposed in [19]. With the improvement of cryptanalysis and design level, it is becoming increasingly difficult to decipher encryption algorithms. However, some algorithms are insecure against various common cryptanalysis methods [20,21,22,23,24,25,26]. Huang et al. [20] presented a simple color image encryption algorithm, in which the permutation process and diffusion process are all related to plaintext. The authors claimed that the algorithm could resist chosen- or known-plaintext attacks efficiently. However, in 2020, Lin et al. [21] found that Huang et al.’s algorithm [20] could not resist chosen-plaintext attacks and they proposed an enhanced algorithm to overcome the flaw. Diab and El-semary [22] broke an image encryption algorithm presented by Chen et al. [23]. An image block encryption algorithm with a sufficient security level and high encryption speed was proposed in [20], while Ma et al. [25] broke the equivalent secret keys successfully by giving five chosen plain images and the corresponding cipher images; Zhu et al. [26] cracked the equivalent key sequence for image obfuscation and image scrambling, respectively, by combining the chosen-plaintext attack and the chosen-ciphertext attack. In [27], Zhu et al. cracked a color image encryption scheme based on combined 1D chaotic maps [28]. An image encryption algorithm using an S-box generated by chaos [29] and a multiple chaotic S-boxes-based image encryption algorithm [30] was broken by Zhu et al. [31] and Lu et al. [32], respectively. It can be found from the literature [20,23,24,29,30] that the main reason why the above algorithms were cracked is that the equivalent key stream of the encryption system had nothing to do with plaintext.

In [33], an image encryption algorithm based on binary bit plane extraction and multiple chaotic maps was proposed, which includes a bit-level permutation for high, 4-bit planes and bit-wise XOR diffusion. It claimed that the algorithm has high security performance. However, the security analysis showed that the key in the bit plane permutation and the key in the diffusion phase are independent of the plain image or the cipher image; therefore, the equivalent diffusion key and the equivalent permutation key can both be obtained by adopting the chosen-plaintext attack. This paper is organized as follows. Section 2 concisely describes the original algorithm in [33]. In Section 3, the security of the algorithm is analyzed, and the equivalent key is cracked by the chosen-plaintext attack method. In Section 4, the experimental simulation is carried out. An improved image encryption algorithm is proposed in Section 5. Section 6 concludes the paper.

## 2. The Original Image Encryption Cryptosystem

This section provides a brief introduction to the original encryption system of [33].

### 2.1. Logistic Chaotic Map and Cubic Logistic Chaotic Map

The logistic chaotic map and the cubic logistic chaotic map are used in the original algorithm. The logistic map is shown as

(1) $$x_{n+1}=\mu_{1}x_{n}\left(1-x_{n}\right)$$

**Figure 1:**
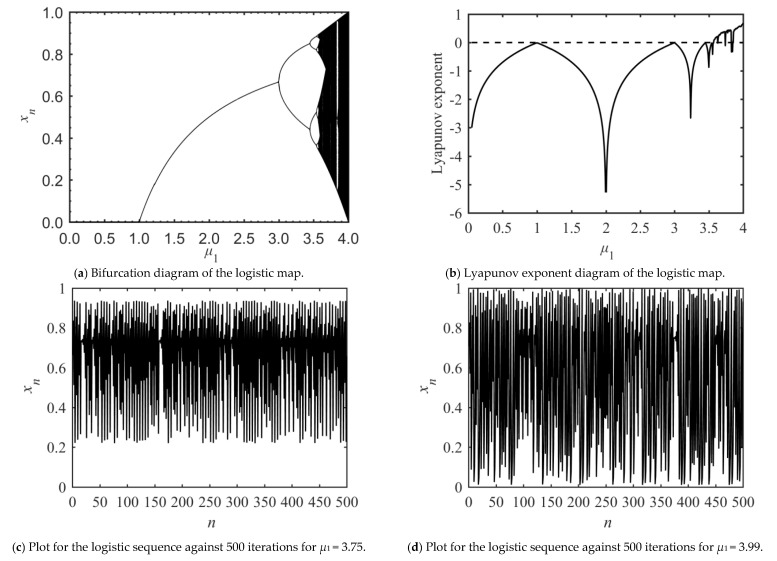
Dynamic system analysis of logistic map.

In order to further determine the value range of parameter *μ*_1_ when the logistic map generates a chaotic sequence, the bifurcation diagram and the Lyapunov exponent diagram of the logistic map are given as Figure 1. It is found that system (1) is chaotic when the control parameter *μ*_1_ ∈ (3.57, 4) and *x_n_* ∈ (0, 1).

The cubic logistic map is defined as

(2) $$y_{n+1}=\mu_{2}y_{n}\left(1-y_{n}\right)\left(2+y_{n}\right)$$

**Figure 2:**
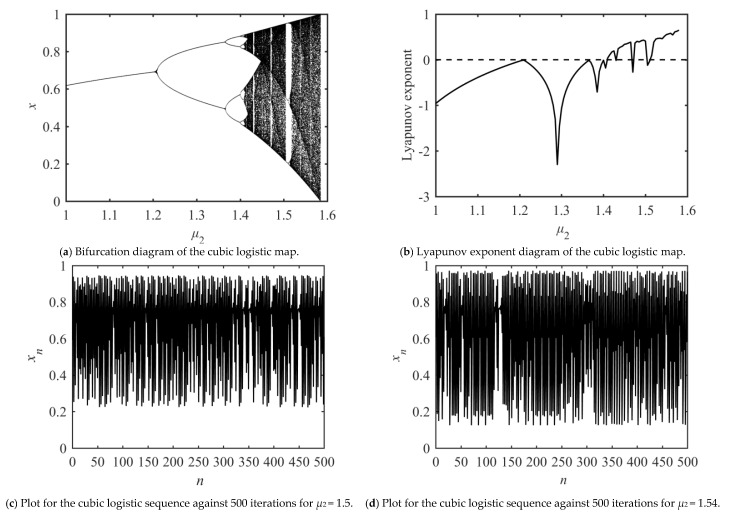
Dynamic system analysis of cubic logistic map.

Similarly, in order to further determine the value range of parameter *μ*_2_ when the cubic logistic map generates a chaotic sequence, the bifurcation diagram and Lyapunov exponent diagram of the cubic logistic map are given as Figure 2. It is found that system (2) is chaotic when the control parameter *μ*_2_ ∈ (1.41, 1.59) and *y_n_* ∈ (0, 1).

### 2.2. Detailed Description of the Original Encryption Algorithm

The secret key of the original encryption algorithm contains four parameters: *x*_0_, $\mu_{1}$, y_0_, $\mu_{2}$. The encryption objects of the original algorithm are a gray image and an RGB color image with size of *H* × *W* (height × width). For the convenience of the description, only the gray image is discussed, and the encryption algorithm of the RGB color image is basically the same. The plain image is defined as P={p(i,j)}, and the permuted image and the cipher image are defined as P′={p′(i,j)} and C={c(i,j)}, respectively. The encryption process includes three stages, as follows:

**Step 1:** Bit plane decomposition. The plain image P={p(i,j)} is decomposed into 8-bit planes $P_{k}=\left\{p_{k}\left(i,j\right)\right\}$ (k = 1, 2,..., 8), given by

(3) $$P=\sum_{k=1}^{8}2^{k-1}p_{k}=p_{1}+2p_{2}+2^{2}p_{3}+2^{3}p_{4}+2^{4}p_{5}+2^{5}p_{6}+2^{6}p_{7}+2^{7}p_{8}$$

Here, let **Z_m_** represent the set [0, *m* − 1], so *p*(*i*, *j*) ∈ **Z**_256_, *p_k_*(*i*, *j*) ∈ **Z**_2_, *P*_1_ and *P*_8_ are the lowest and highest bit planes, respectively;

**Step 2:** Bit-level permutation. The permutation process is only for the high, 4-bit planes, the 8-th bit plane is described as an example. Firstly, given the initial value y_0_ and the control parameter $\mu_{2}$, the cubic logistic map is iterated to get two real sequences, {*y*_1_, *y*_2_,…, *y*_H_} with length *H* and {*y*_H+1_, *y*_H+2_,…, *y*_H+W_} with length *W*, respectively. Then, the two real number sequences are sorted in ascending order to obtain the position index sequence $RS={\left\{rs\left(i\right)\right\}}_{i=1}^{H}$ and $CS={\left\{cs\left(j\right)\right\}}_{j=1}^{W}$, respectively. Then, using the two sequences *RS* and *CS*, the permutation bit plane $P_{8}\prime ={\left\{p_{8}\prime \left(i,j\right)\right\}}_{i=1,\;j=1}^{H,\;W}$ corresponding to the original 8-th bit plane $P_{8}={\left\{p_{8}\left(i,j\right)\right\}}_{i=1,j=1}^{H,W}$ is obtained by Equation (4)

(4) $$p_{8}\prime \left(i,j\right)=p_{8}\left(rs(i),cs(j)\right)$$

Similarly, through Equation (4), the permuted bit planes $P_{5}\prime ,P_{6}\prime ,P_{7}\prime$ can be obtained from $P_{5},P_{6},P_{7}$, respectively. Finally, the permuted image P′ is obtained though Equation (5)

(5) $$P\prime =P_{1}+2P_{2}+2^{2}P_{3}+2^{3}P_{4}+2^{4}P_{5}\prime +2^{5}P_{6}\prime +2^{6}P_{7}\prime +2^{7}P_{8}\prime$$

where $p_{1},p_{2},p_{3},p_{4}$ are the low 4-bit planes, and $p_{5}\prime ,p_{6}\prime ,p_{7}\prime ,p_{8}\prime$ are the high 4-bit planes, respectively;

**Step 3:** Bit-wise XOR diffusion. Firstly, setting x_0_ and $\mu_{1}$ as the initial value and control parameter of the logistic map, respectively, a real matrix $R={\left\{r\left(i,j\right)\right\}}_{i=1,j=1}^{H,W}$ is obtained by iterating the logistic map *H* × *W* times. Then, a mask image $M={\left\{m\left(i,j\right)\right\}}_{i=1,j=1}^{H,W}$ is obtained by Equation (6)

(6) $$m\left(i,j\right)=\mathrm{mod}\left(floor\left(r\left(i,j\right)\times 10^{5}\right),256\right)$$

Then, through Equation (7), the ciphertext $C={\left\{c\left(i,j\right)\right\}}_{i=1,j=1}^{H,W}$ can be obtained as

(7) c(i,j)=p′(i,j)⊕m(i,j)

It can be seen that the key set of the encryption system in [33] is keys = {*μ*_1_, *x*_n_, *μ*_2_, *y_n_*}. If we choose an accuracy of 10^−14^ for the four variables (*μ***_1_**, *x***_0_**, *μ***_2_**, *y*_0_), we obtain a key space of 10^56^ ≈ 2^187^. As [34,35,36] pointed out, the effective key space of the image encryption system should be greater than 2^100^ in order to prevent brute force attacks, so the key space of our algorithm is sufficiently large to resist against brute force attacks.

## 3. Security Analysis of the Original Algorithm and Chosen-Plaintext Attack

Through the security analysis, we found that the encryption system has the following security defects:

(1) The chaotic sequences used for encryption are independent of the plaintext image. In other words, when the keys are fixed, the chaotic sequences used for encryption are unchanged for different plaintext images of the same size;
(2) The diffusion part is too simple, as only XOR diffusion is adopted, in which neither a nonlinear function nor a complicated diffusion mechanism is involved. Therefore, the algorithm is not sensitive to plain images;
(3) Permutation and diffusion are independent of each other, and there is no relationship between them. Therefore, the permutation and diffusion parts of the original algorithm can be deciphered by the strategy of divide and conquer.

From the encryption process of the original algorithm, it can be found that two sequences, RS and CS, are used in the scrambling process, and the chaotic sequence, M, is used in the diffusion phase. Therefore, the equivalent key streams of the original algorithm are M, RS and CS. If the equivalent key streams are cracked, the original encryption system will be cracked.

The so-called chosen-plaintext attack and selective plaintext attack refer to the following process. In addition to not knowing the secret keys used by the cryptosystem, the attacker understands the working mechanism of the encryption algorithm and has the opportunity to use the encryption machine of the cryptosystem. Therefore, the attacker can choose some special plaintext images and obtain the corresponding ciphertext images, thereby deciphering the equivalent secret keys of the cryptosystem or the target ciphertext image.

### 3.1. Cracking of Equivalent Key M in the Diffusion Phase

For bit-level permutation, if all the bits of the input plaintext image are the same, that is, all are 0 or all are 1, then the corresponding permutation image is exactly the same as the plain image. For example, by choosing the image $P_{0}={\left\{p_{0}\left(i,j\right)=0\right\}}_{i=1,j=1}^{H,W}$, whose pixel values are all 0, as the input plain image, the result, $P_{0}\prime$, after bit-level permutation is exactly the same as the original plain image, that is $P_{0}\prime =P_{0}$. The attacker obtains the cipher image $C_{0}={\left\{c_{0}\left(i,j\right)\right\}}_{i=1,j=1}^{H,W}$ corresponding to P_0_. Finally, according to formula (7), the attacker can obtain $M={\left\{m\left(i,j\right)\right\}}_{i=1,j=1}^{H,W}$ as

(8) $$m\left(i,j\right)=p\prime \left(i,j\right)⊕c_{0}\left(i,j\right)=0⊕c_{0}\left(i,j\right)=c_{0}\left(i,j\right)$$

Therefore, the equivalent key *M* is cracked in the diffusion phase.

### 3.2. Breaking Bit-Level Permutation

From the permutation of Formula (4), we find that the essence of permutation is to exchange the rows and columns of the bit plane matrix. After permutation, the elements of the same row are still in the same row, and the elements of the same column are in the same column.

For example, taking a plain image, P, of size 4 × 4 as an example, let *RS* = [3, 2, 4, 1], *CS* = [2, 1, 4, 3], and

(9) $$P=\left[\begin{array}{cccc} 1 & 2 & 3 & 4\\ 5 & 6 & 7 & 8\\ 9 & 10 & 11 & 12\\ 13 & 14 & 15 & 16 \end{array}\right]$$

Then, the permuted image, P′, is obtained through Formula (4)

(10) $$P\prime =\left[\begin{array}{cccc} 10 & 9 & 12 & 11\\ 6 & 5 & 8 & 7\\ 14 & 13 & 16 & 15\\ 2 & 1 & 4 & 3 \end{array}\right]$$

In the original algorithm, the bit plane whose element was 0 or 1 is permuted, so the sequences, *RS* and *CS*, can be recovered by constructing a special bit plane. Taking the 8-th bit plane as an example, a special plain image is constructed so that its 8-th bit plane has the following form:

(11) $$P_{8}={\left[\begin{array}{cccccc} 1 & 0 & 0 & 0 & \ldots & 0\\ 1 & 1 & 0 & 0 & \ldots & 0\\ 1 & 1 & 1 & 0 & \ldots & 0\\ \ldots & \ldots & \ldots & \ldots & \ldots & \ldots \\ 1 & 1 & 1 & 1 & \ldots & 1 \end{array}\right]}_{H\times W}$$

Because the numbers of element 1 in each row (column) of $P_{8}$ are different, the sequences, RS and CS, can be obtained by comparing the numbers of element 1 in each row (column) in the 8-th bit plane $P_{8}\prime$ of the permuted image, P′. Suppose H = 4, W = 4, and then the scrambled $P_{8}\prime$ is

$$P_{8}\prime =\left[\begin{array}{cccc} 1 & 1 & 0 & 1\\ 1 & 1 & 0 & 0\\ 1 & 1 & 1 & 1\\ 0 & 1 & 0 & 0 \end{array}\right]$$

Based on $P_{8}\prime$, it can be inferred that *RS* = [3, 2, 4, 1] and *CS* = [2, 1, 4, 3].

### 3.3. Specific Steps of Chosen-Plaintext Attack

The specific steps of our chosen-plaintext attack are as follows:

**Step1**: The chosen-plaintext attack means that the attacker has the access right of the encryptor and can construct the ciphertext corresponding to any plaintext. Thus, as shown in Section 3.1, by choosing the all-zero image, *P*_0_, as the input plain image, one gets the corresponding cipher image, *C*_0_, then *M* = *C*_0_;

**Step 2:** Select a special plaintext image so that its 8-th bit plane has the form of matrix (11) and so the selected plain image can be the following matrix image, PP

(12) $$PP={\left[\begin{array}{cccccc} 128 & 0 & 0 & 0 & \ldots & 0\\ 128 & 128 & 0 & 0 & \ldots & 0\\ 128 & 128 & 128 & 0 & \ldots & 0\\ \ldots & \ldots & \ldots & \ldots & \ldots & \ldots \\ 128 & 128 & 128 & 128 & \ldots & 128 \end{array}\right]}_{H\times W}$$

After encryption, obtain the cipher image, CP, corresponding to PP, then obtain the permuted image PP′ of PP is by using the cracked diffusion key M, the elements of which are shown in Equation (13)

(13) $$pp\prime \left(i,j\right)=c_{0}\left(i,j\right)⊕m\left(i,j\right)$$

**Step 3:** Extract the 8-th bit plane $PP_{8}\prime$ of PP′, as shown in Section 3.2. By comparing the numbers of element 1 in each row of $PP_{8}\prime$, the vector RS is obtained. Similarly, the vector, *CS*, is also obtained by comparing the numbers of element 1 in each column of $PP_{8}\prime$;

**Step 4:** As for a given cipher image, *C*, firstly the permuted image, P′, is obtained by using the diffusion key *M* though Equation (14)

(14) p′(i,j)=c(i,j)⊕m(i,j)

Extract the 5-8th bit planes of P′, then perform reverse permutation on the 5-8th bit planes of P′ to obtain the 5-8th bit planes of the plaintext image P by using the sequences *CS* and *RS*. The 1-4th bit planes of *P* are exactly the same as that of P′. In this way, all the eight-bit planes of P are obtained, and then the plain image P can be obtained by Formula (3).

### 3.4. The Discussion

In [37], the algorithm in [33] is cracked, but for an 8-bit grayscale image of size 256 × 256, the data complexity of the attack method required for breaking the algorithm is O(log_2_(*H* × *W*)) = O(19), while only one special plaintext image and its corresponding ciphertext image is needed to decode the scrambling sequences, *RS* and *CS*, in our method. As such, the complexity of our attack algorithm is greatly reduced.

## 4. Experimental Simulations of Cracking

**Figure 3:**
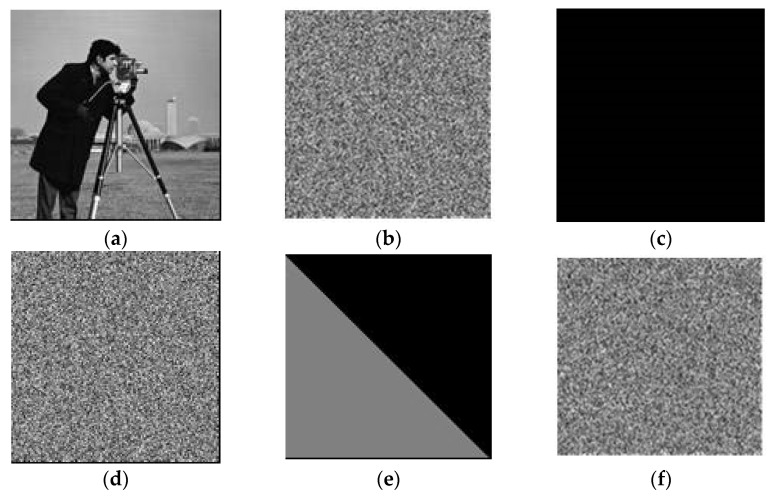
**The** results of the test images. (**a**) “Cameraman” plain image. (**b**) The encrypted “Cameraman”. (**c**) All black plain image. (**d**) Encrypted “black image”. (**e**) The special image as Formula (12). (**f**) The cipher image of (**e**).

**Figure 4:**
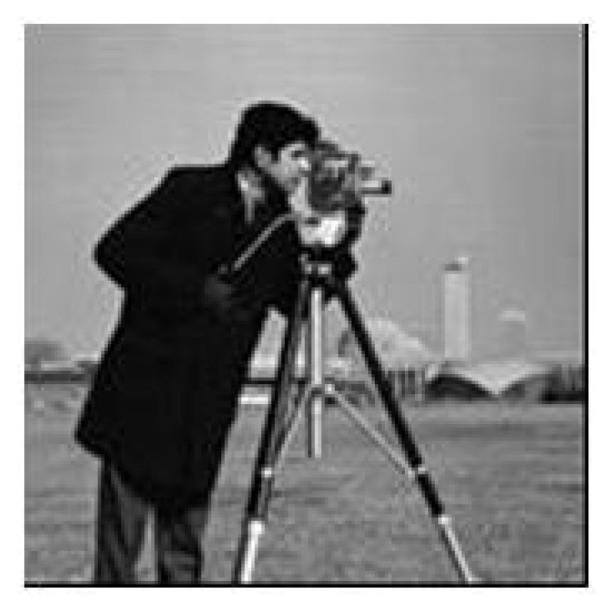
The decrypted image.

The experimental image is an 8-bit grayscale image, Cameraman, of size 256 × 256. The process is as follows: choose keys x_0_ = 0.8578, $\mu_{1}$ = 3.6832, y_0_ = 0.3476, $\mu_{2}$ = 1.5866; encrypt the image, Cameraman, size 256 × 256, with a pixel value of 0, and a special image of size 256 × 256 as Formula (12) to obtain the corresponding encrypted image, as shown in Figure 3b,d,f. According to Figure 3d, the mask matrix, M, can be decrypted without knowing the key. The permutation sequences, *RS* and *CS*, can be decrypted by combining the matrix, M, and Figure 3f. Therefore, all the equivalent keys can be decrypted. Furthermore, any encrypted image can be decrypted by using the equivalent keys. After attacking the encrypted image, Figure 3b, the recovered image is shown in Figure 4.

## 5. The Improved Algorithm and Security Analysis

The main reason why the original algorithm is cracked is that the equivalent keys M, RS and CS of the original encryption algorithm are independent of the plain image. According to the five suggestions given in [37], we propose the improved algorithm of [33]. The key set of the improved algorithm is exactly the same as that of the original algorithm. Compared with the original algorithm of [33], the improved algorithm can resist chosen-plaintext attack and has better security performance.

### 5.1. The Improved Encryption Algorithm

The specific steps of the improved encryption algorithm are as follows:

**Step 1:** In the permutation phase, the initial value y_0_ of chaotic map (2) is related to the sum of the pixel values of the 8-th bit plane of the plain image

(15) $$sum=\sum_{i=1}^{H}\sum_{j=1}^{W}p_{8}\left(i,j\right)$$

Then, y_0_ is updated with sum:

(16) $$y_{0}=(\mathrm{mod}(floor(y_{0}\times 100\times sum),W\times H))/(W\times H)$$

In this way, the two permutation sequences, RS and CS, will be related to the plain image. The permutation process is exactly the same as the original algorithm;

**Step 2:** The permuted image, P′={p′(i,j)}, and the real matrix, R={r(i,j)}, of the original algorithm are all transformed into one-dimensional vector sequences P′={p′(1), p′(2),⋯, p′(H×W)} and $R=\left\{\begin{array}{cccc} r(1), & r(2), & \ldots , & r(H\times W) \end{array}\right\}$, respectively shown from left to right and from top to bottom. The ciphertext sequence *C* = {*c*(1), *c*(2), …, *c*(*H* × *W*)} is generated according to Formulas (17)–(21), then sequence *C* is transformed into a matrix of size *H* × *W* to obtain the final cipher image;

(17) $$m\left(1\right)=\mathrm{mod}\left(floor\left(r\left(1\right)\times 10^{5}\right),256\right)$$

(18) c(1)=p′(1)⊕m(1)

(19) $$m\left(i\right)=\mathrm{mod}\left(floor\left(r\left(i\right)\times c\left(i-1\right)\times 10^{5}\right),256\right)$$

(20) kt(i)=floor(m(i)×(i−1)/256)+1

Obviously, *kt*(*i*) ∈ [1, *i* − 1]. *i* = 2, 3, 4,…, *H* × *W*.

(21) c(i)=mod(p′(i)+m(i),256)⊕c(kt(i))

It can be seen from Equation (19) that the generation of the key, *M*, is related to the cipher image, so the key, *M*, used for encrypting different plaintext is different. Therefore, the improved algorithm can resist a chosen-plaintext attack. Furthermore, the ciphertext feedback mechanism is adopted in the Formulas (20) and (21), which overcomes the weakness that the original algorithm is not sensitive to the plain image.

### 5.2. The Improved Decryption Algorithm

The specific steps of the improved decryption algorithm are as follows:

**Step 1**: Transform the ciphertext image matrix to a sequence *C* = {*c*(1), *c*(2), …, *c*(*H* × *W*)}, and the real chaotic sequence *R* ={*r*(1), *r*(2), …, *r*(*H* × *W*)};

**Step 2**: Decrypt the first pixel value *c*(1) to obtain the first pixel value *p*’(1) by

(22) $$\{\begin{array}{l} m(1)=\mathrm{mod}(\mathrm{floor}(r(1)\times 10^{5}),256)\\ p\prime (1)=\mathrm{bitxor}(m(1),c(1)) \end{array}$$

**Step 3**: Decrypt the i-th pixel value *C*(*i*) to obtain the i-th pixel value *P*’(*i*) by

(23) $$\{\begin{array}{l} \begin{array}{l} m(1)=\mathrm{mod}(\mathrm{floor}(r(i)\times c(i-1)\times 10^{5}),256)\\ kt(i)=\mathrm{floor}(m(i)\times (i-1)/256)+1 \end{array}\\ p\prime (i)=\mathrm{mod}(\mathrm{bitxor}(c(i),c(kt(i)))-m(i),256) \end{array}$$

where *i* = 1,2,..., *H* × *W*;

**Step 4**: Transform the 1D sequence *P*’ = [*p*’(1), *p*’(2),..., *p*’(*H* × *W*)] to a matrix size of *H* × *W* as *P*’ = {*p*’(i, j)|*i* =1, 2,..., *H*, *j* = 1, 2,..., *W*};

**Step 5**: Bit plane decomposition. Decompose the intermediate version image $P\prime ={\left\{p\prime \left(i,j\right)\right\}}_{i=1,j=1}^{H,W}$ with the size of *H* × *W* into 8-bit planes, *PP*_1_ to *PP*_8_, and calculate the sum of pixel values of the 8-th bit plane of the binary image *PP*_8_

(24) $$sum=\sum_{i=1}^{H}\sum_{j=1}^{W}pp_{8}\left(i,j\right)$$

The result of the above equation is equal to the result of Equation (15);

**Step 6**: Calculate y_0_ with Equation (16), and generate the two permutation sequences, *RS* and *CS*, by using the same method as the original algorithm;

**Step 7**: Perform an inverse scrambling operation on the planes of (*PP*_5_, *PP*_6_, *PP*_7_, *PP*_8_) to obtain the planes of (*P*_5_, *P*_6_, *P_7_*, *P*_8_) by using *RS* and *CS* as:

*p_5_(rs(i, j), cs(i, j)) = pp_5_(i, j), p_6_(rs(i, j), cs(i, j)) = pp_6_(i, j), p_7_(rs(i, j), cs(i, j)) = pp_7_(i, j), p_8_(rs(i, j), cs(i, j)) = pp_8_(i, j). i =1, 2,..., H, j = 1, 2,..., W.*

**Step 8**: Combine 8-bit planes to obtain the restored original image, *P*, by

(25) $$P=2^{7}\times P8+2^{6}\times P7+2^{5}\times P6+2^{4}\times P5+2^{3}\times PP4+2^{2}\times PP3+2\times PP2+PP1$$

### 5.3. Analysis of Improved Algorithm to Resist Chosen-Plaintext Attack

The improved algorithm can resist the chosen-plaintext attack, which is reflected in two aspects. Firstly, from Formulas (15) and (16), it can be seen that the scrambling sequences, *RS* and *CS*, produced in the scrambling stage are related to the plaintext image, and the sequences, *RS* and *CS*, used to encrypt the different images are different. Secondly, from Equations (19) and (20), we can see that the generation of m(i) is related to the previous ciphertext value c (i − 1), and the generation of kt(i) is related to m(i). Therefore, the sequences, m(i) and kt(i), used to encrypt different images are different. In short, the improved algorithm has the effect of “one-time pad”.

### 5.4. Comparison of Ciphertext Security Performance between Improved Algorithm and Original Algorithm

In order to further highlight the advantages of the improved algorithm, we will compare it with the original algorithm from the aspects of information entropy, ciphertext correlation analysis and ciphertext sensitivity.

(1) Comparison of Information Entropy

The ideal value of entropy for an 8-bit gray-scale image is 8. The closer the value is to 8, the more uncertain the image is, and the more uniform the distribution of image pixel value is. Table 1 shows the information entropy of the cipher images Rice, Cameraman, Lena and Pepper, which were encrypted by the improved algorithm and the original algorithm. Compared to the original algorithm and other algorithms, the improved algorithm is closer to the ideal situation, that is, the encryption effect of this algorithm is better.

(2) Comparison of Correlation Coefficient

In general, there is a strong correlation between the adjacent pixels of the plaintext image, while the correlation between the adjacent pixels of the ciphertext image is close to zero. Table 2 shows the correlation coefficient of the cipher images of Cameraman and Peppers encrypted by the improved algorithm and the original algorithm in the horizontal direction, the vertical direction and the diagonal direction, respectively.

(3) Comparison of Plaintext Sensitivity

The number of pixels change rate (NPCR) and unified average changing intensity (UACI) are commonly used to measure the sensitivity of encryption algorithms to plaintext. The formulas for the calculation of NPCR and UACI are found in [10]. For the 256 levels of the grayscale images, the expected values of NPCR and UACI are 99.6094% and 33.4635%, respectively.

We have performed 20 groups of tests. In each test, we randomly selected one pixel in the plain image Cameraman, changed its value with 1 bit and encrypted it. Finally, we calculated the NPCR and UACI values between any two pairs of ciphertext image. The results are shown in Table 3. From Table 3, one can see that the NPCR and UACI values are very close to the ideal values in the improved algorithm, while in the original algorithm, the values of NPCR and UACI are close to 0. This is mainly because the original algorithm does not adopt the ciphertext feedback mechanism in the diffusion stage, so the original algorithm is not sensitive to plaintext.

## 6. Conclusions

In this paper, the security performance of a recent chaotic image encryption cryptosystem based on bit planes extraction and multiple chaotic maps is cryptanalyzed in detail. It is found that the equivalent key streams *M*, *RS* and *CS* can be recovered separately in the scenario of a chosen-plaintext attack. In order to overcome the shortcomings of the original algorithm, which cannot resist the chosen-plaintext attack and is not sensitive to plaintext, we propose an improved encryption algorithm. The innovation of the improved algorithm lies in that the key set of the encryption system is the same as that of the original algorithm, but the equivalent sequences, *M*, *RS* and *CS*, used to encrypt different images, are different, which has the effect of a one-time pad.

The improved algorithm has the advantages of high security and resistance to chosen-plaintext attacks. However, it also has the following defects: from Formulas (19) and (20), we can see that in the encryption process, we need to switch back and forth between the floating-point operation and the integer operation (that is, one floating-point operation, one integer operation, switching back and forth), which is not conducive to hardware implementation. Therefore, it is still necessary to design a secure and efficient image encryption algorithm based on chaos.

## Figures and Tables

**Table 1:** Information entropy of ciphertext image.

Images	The Improved Algorithm	The Original Algorithm	Ref. [38]	Ref. [39]
Cameraman	7.9972	7.8716	7.9921	7.9987
Rice	7.9973	7.8921	7.9945	7.9965
Pepper	7.9990	7.8608	7.9934	7.9929
Lena	7.9989	7.8769	7.9968	7.9973

**Table 2:** Correlation coefficient analysis of two adjacent pixels.

The Test Image	Direction	The Improved Algorithm	The Original Algorithm
Cameraman	Horizontal	0.0266	0.2237
Cameraman	Vertical	−0.0088	−0.0504
Cameraman	Diagonal	−0.0049	−0.0131
Peppers	Horizontal	0.0078	−0.0490
Peppers	Vertical	0.0148	0.3377
Peppers	Diagonal	0.0113	−0.0318

**Table 3:** Comparison of plaintext sensitivity between the improved algorithm and the original algorithms.

Algorithms	NPCR (%)		UACI (%)	
	Min	Average	Min	Average
The improved algorithm	99.48	99.68	32.43	33.36
The original algorithm	0	0.00001	0	0.0000001
Ref. [10]	99.53	99.67	33.52	33.64

## Data Availability

Data sharing not applicable.
